# Cannabidiol as a Promising Adjuvant Therapy for Estrogen Receptor-Positive Breast Tumors: Unveiling Its Benefits with Aromatase Inhibitors

**DOI:** 10.3390/cancers15092517

**Published:** 2023-04-27

**Authors:** Cristina Ferreira Almeida, Natércia Teixeira, Maria João Valente, Anne Marie Vinggaard, Georgina Correia-da-Silva, Cristina Amaral

**Affiliations:** 1 UCIBIO/REQUIMTE, Laboratory of Biochemistry, Department of Biological Sciences, Faculty of Pharmacy, University of Porto, Rua Jorge Viterbo Ferreira, n° 228, 4050-313 Porto, Portugal; 2 Associate Laboratory i4HB—Institute for Health and Bioeconomy, Faculty of Pharmacy, University of Porto, Rua Jorge Viterbo Ferreira, n° 228, 4050-313 Porto, Portugal; 3 National Food Institute, Technical University of Denmark, 2800 Kongens Lyngby, Denmark

**Keywords:** breast cancer, aromatase inhibitors, anastrozole, letrozole, exemestane, cannabinoids, cannabidiol, aromatase, estrogen receptor, androgen receptor

## Abstract

**Simple Summary:**

Estrogen receptor-positive (ER^+^) breast cancer is the most prevalent breast cancer subtype, accounting for 70–85% of all cases. The combination of endocrine therapy, such as aromatase inhibitors (AIs), with target therapy is one of the most recent approaches, but its effectiveness is not optimal. Cannabidiol (CBD) has demonstrated important anti-tumor effects on ER^+^ breast cancer cells. Considering this, our goal was to evaluate the effects of combining CBD with the AIs currently in use in the clinical context. Our results revealed that CBD may be particularly beneficial when combined with the AI exemestane (Exe), since it potentiates the anti-tumor effects of Exe through the modulation of cell death and specific targets, including ERα and androgen receptor (AR). This reinforces the beneficial potential of cannabinoids in breast cancer and points to the possibility of improving Exe effects through an adjuvant therapy with CBD.

**Abstract:**

Background: Estrogen receptor-positive (ER^+^) breast cancer is the most diagnosed subtype, with aromatase inhibitors (AIs) being one of the therapeutic drug types used in the clinic. However, endocrine resistance may develop after prolonged treatment, and different approaches, such as combining endocrine and targeted therapies, have been applied. Recently, we demonstrated that cannabidiol (CBD) induces anti-tumor actions in ER^+^ breast cancer cells by targeting aromatase and ERs. Considering this, we studied, in vitro, whether CBD when combined with AIs could improve their effectiveness. Methods: MCF-7aro cells were used and the effects on cell viability and on the modulation of specific targets were investigated. Results: CBD when combined with anastrozole (Ana) and letrozole (Let) caused no beneficial effect in comparison to the isolated AIs. In contrast, when combined with the AI exemestane (Exe), CBD potentiated its pro-cell death effects, abolished its estrogen-like effect, impaired ERα activation, and prevented its oncogenic role on the androgen receptor (AR). Moreover, this combination inhibited ERK_1/2_ activation, promoting apoptosis. The study of the hormonal microenvironment suggests that this combination should not be applied in early stages of ER^+^ breast tumors. Conclusions: Contrary to Ana and Let, this study highlights the potential benefits of combining CBD with Exe to improve breast cancer treatment and opens up the possibility of new therapeutic approaches comprising the use of cannabinoids.

## 1. Introduction

The latest data regarding cancer statistics show that female breast cancer is the most frequently diagnosed cancer worldwide, as well as the leading cause of cancer-related death in women [1,2]. Among the various breast cancer subtypes, the most common tumors are estrogen receptor-positive (ER^+^) [1,3], which make them suitable for endocrine therapy, namely with the aromatase inhibitors (AIs), and the anti-estrogens tamoxifen and fulvestrant. In fact, the third-generation of AIs, which includes anastrozole (Ana), letrozole (Let) and exemestane (Exe), is the standard treatment for post-menopausal women and for pre-menopausal women after ovarian function suppression [3,4,5,6,7]. In general, several studies using different MCF-7 derivative cell lines have demonstrated that the anti-cancer properties of AIs rely on the disruption of cell cycle progression, promotion of apoptosis through the involvement of the mitochondrial pathway, or caspase-6 activation and reduced levels of the anti-apoptotic proteins from the Bcl-2 family, as well as the induction of cellular senescence [8,9,10,11]. Furthermore, the steroidal AI Exe also induces a cytoprotective autophagic process in MCF-7aro cells [8] that blocks the occurrence of senescence, a process that has been recently associated with the non-steroidal AIs Ana and Let [9]. In addition, our group studied the effects of the hormonal environment on AIs actions in MCF-7aro cells and found that Exe, besides acting as an AI, also modulates ERα and AR, presenting a weak estrogen-like behavior [9] and an oncogenic effect through the AR [12], while Ana and Let act as pure AIs [9]. Besides the therapeutic success of the third-generation of AIs, these compounds may induce some undesired side effects, and their prolonged use may lead to the development of endocrine resistance, which is of major clinical concern [7]. In order to surpass this issue, combined endocrine therapy and modulators of several signaling pathways, such as CDK4/6 inhibitors, mTOR inhibitors, PI3K inhibitors, or AR antagonists, have been applied through recent years [4,13,14,15,16]. In fact, the latest guidelines suggest the use of CDK4/6 inhibitors combined with AIs or fulvestrant as the standard therapy for ER^+^ advanced breast cancer in both pre- and post-menopausal women [4]. However, these combinations may induce undesired side effects and their success has been limited, as they do not improve the overall survival [7,16,17]. Furthermore, it is known that 10% of patients using CDK4/6 inhibitors therapy may develop de novo resistance, while others may develop acquired resistance after 24–28 months with first-line therapy or after a shorter period with second-line therapy [18,19]. Thus, the search for alternative therapeutic approaches remains imperative.

Cannabinoids have been known for their multiple beneficial effects for a long time, and their clinical interest has been rising during recent years with some cannabinoid-based medicines already being approved for clinical use [20,21]. These compounds have been investigated in the context of various diseases and conditions, including pain, epilepsy, asthma, sleep disorders, depression, inflammation, and cancer, and for the relief of chemotherapy-related side effects [22,23,24,25]. Besides their anti-emetic effects, cannabinoids have already demonstrated, in vitro and in vivo, important and promising anti-tumor properties in different types of cancer. Anti-proliferative, anti-angiogenic, anti-invasive, and anti-metastatic effects are some of the actions attributed to cannabinoids [26,27,28,29,30,31,32,33]. These beneficial effects are supported by the fact that multiple pathological conditions, including cancer, present alterations in the endocannabinoid system, and that cannabinoid signaling is known to interact with other signaling pathways involved in cell growth, differentiation, metabolism, and apoptosis, such as AKT, EGFR, and mTOR [34]. In addition, it is known that the endocannabinoid system plays a role in the development and aggressiveness of breast cancer. The cannabinoid receptor CB2 is positively correlated with ER status and negatively correlated with tumor grade, whereas the expression of the cannabinoid receptor CB1 is positively correlated with tumor grade [35].

In relation to breast cancer, most of the studies conducted so far were performed on triple-negative breast tumors, whereas studies in ER^+^ and human epidermal growth factor receptor 2 positive (HER2^+^) tumors are still scarce [29,36,37]. Previous work from our group revealed that the phytocannabinoids cannabidiol (CBD) and Δ^9^-tetrahydrocannabinol (THC), and the endocannabinoid anandamide (AEA), are able to induce MCF-7aro cell cycle arrest and modulate aromatase and ERs (ERα and ERβ), the main therapeutic targets in ER^+^ breast tumors, highlighting their pharmacological potential [38]. Despite that, only CBD and AEA were able to inhibit aromatase in human placental microsomes [39,40]. Moreover, we also demonstrated that, among the cannabinoids studied, the most promising one was CBD. It induced the most pronounced anti-proliferative effects by acting as a multi-target molecule, inhibiting and decreasing aromatase expression, down-regulating ERα and up-regulating ERβ expression, all therapeutic advantages from a clinical point of view [38]. Considering all this, in this work, our goal was to evaluate, in vitro, whether CBD when combined with the AIs under clinical use could render a beneficial adjuvant therapy. Moreover, we also intended to investigate whether the hormonal environment can influence the behavior of these combinations.

## 2. Materials and Methods

### 2.1. Cell Culture

The effects of CBD in combination with the AIs were evaluated on an ER^+^ breast cancer cell line that overexpresses aromatase, MCF-7aro cells, kindly provided by Dr. Shiuan Chen (Beckman Research Institute, City of Hope, Duarte, CA, USA). Since these cells express high levels of aromatase, they are considered a good in vitro cell model to study this type of breast cancer [10]. Moreover, in order to address possible cytotoxic effects of the combinations on non-tumoral cells, the human foreskin fibroblast cell line (HFF-1) was also used (ATCC, Manassas, VA, USA). Both cell lines were cultured at 37 °C and in a 5% CO_2_ atmosphere.

The HFF-1 cells were cultured in a DMEM (Gibco Invitrogen Co., Paisley, Scotland, UK) glucose-enriched medium without phenol-red and supplemented with 1 mM of sodium pyruvate, 1% of penicillin–streptomycin–amphotericin B, 2 mM of L-glutamine, and 10% of FBS. For MCF-7aro cells, Eagles’s minimum essential medium (MEM; Gibco Invitrogen Co., Paisley, Scotland, UK) with phenol-red, 10% heat-inactivated FBS, 1 mM of sodium pyruvate, 1% of penicillin–streptomycin–amphotericin B, and 100 μg/mL of Geneticin (G418) were used (Gibco Invitrogen Co., Paisley, Scotland, UK). In order to avoid the interference of the hormones present in FBS and the estrogen-like properties of phenol-red [41], cells were cultured in a estrogen-free MEM without phenol-red and supplemented with 5% of pre-treated charcoal heat-inactivated fetal bovine serum (CFBS), 1 mM of sodium pyruvate, 1% of penicillin–streptomycin–amphotericin B, and 2 mM of L-glutamine (Gibco Invitrogen Co., Paisley, Scotland, UK)) three days before the beginning of the experiments. All MCF-7aro experiments were performed in these conditions and in the presence of 1 nM of testosterone (T), used as an aromatase substrate and proliferation inducing agent [8], or with 1 nM of estradiol (E_2_; Sigma-Aldrich Co., Saint Louis, MI, USA), the product of the aromatization reaction [8].

The stock solutions of CBD (Tocris Bioscience, Biogen Cientifica, S.L., Spain), Ana, Let, Exe (Carbosynth, Berkshire, UK), ICI 182780 (Fulvestrant; ICI), and Casodex (Bicalutamide; CDX; Sigma-Aldrich Co., Saint Louis, MI, USA) were prepared in 100% DMSO (Sigma-Aldrich Co., Saint Louis, MI, USA) and stored at −20 °C. The stock solutions of T and E_2_ were prepared in absolute ethanol and also stored at −20 °C. The compounds were diluted in culture medium before each experiment, with the final concentrations of DMSO and ethanol being lower than 0.05%. Additionally, all the controls used for each experiment contained the vehicles in these conditions.

### 2.2. Cell Viability

To determine the effect of the AIs, CBD, and their combinations on cell viability, we used the 3-(4,5-dimethylthiazol-2-yl)-2,5-difenyltetrazolium (MTT) and the lactate dehydrogenase (LDH) release method. To perform these assays, cells were cultured in 96-well plates at a cellular density of 7.5 × 10^3^ cells/mL (6 days) for HFF-1 cells, and at 2 × 10^4^ cells/mL (3 days) and 1 × 10^4^ cells/mL (6 days) for MCF-7aro cells. Cells were incubated with Ana, Let, and Exe at 10 µM, CBD (1 and 5 µM), and their combinations. Regarding MCF-7aro cells, they were also treated with 1 nM of T or E_2_, and the cells treated only with T or E_2_ were considered as the control. For HFF-1 cells, cells treated only with the medium were designated as the control. In both cases, the controls represent the maximum of cell viability (100%).

After treatment, 3-(4,5-Dimethylthiazol-2-yl)-2,5-diphenyltetrazolium bromide (MTT) (0.5 mg/mL) (Sigma-Aldrich Co., Saint Louis, MO, USA) was added and viability was quantified spectrophotometrically in a Biotek Sinergy HTX Multi-Mode Microplate Reader (Biotek Instruments, Winowski, VT, USA), while the LDH release assay was performed with 10% of the culture medium of each well using the CytoTox 96 nonradioactive cytotoxicity assay kit (Promega Corporation, Madison, WI, USA), according to the manufacturer’s protocol. All the experiments were performed in triplicate in at least three independent experiments. The results are expressed as the relative percentage of the control cells.

### 2.3. Analysis of Apoptosis

In order to investigate whether the anti-proliferative effects of the combinations of AIs with CBD were a result of an apoptotic process in the MCF-7aro cells, the activities of caspase-7, 8, and 9 were measured as previously reported [42]. The experiments were performed using a luminescent assay with Caspase-Glo^®^ 3/7, Caspase-Glo^®^ 8, and Caspase-Glo^®^ 9, according to the manufacturer’s instructions (Promega Corporation, Madison, WI, USA). Cells were plated on a 96-well white plate at a cell density of 2 × 10^4^ cells/mL, and incubated with the compounds in the presence of 1 nM of T or E_2_ for 2 days. Cells treated with 10 µM staurosporine (STS, Sigma-Aldrich Co., Saint Louis, MO, USA) were used as a positive control.

Luminescence was measured using a Biotek Synergy HTX Multi-Mode Microplate Reader (Biotek Instruments, Winowski, VT, USA). The results are expressed relative to untreated control cells and data are presented as relative luminescence units (RLU). All the assays were performed in triplicate in at least three independent experiments.

### 2.4. Western Blot Analysis

To perform Western blot analysis, MCF-7aro cells were plated in 6-well plates (7.5 × 10^5^ cells/mL) and treated with the AIs (10 µM), CBD (1 and 5 µM) and their combinations, with or without ICI (100 nM) or CDX (1 µM), over 3 days. Cells treated only with 1 nM T were used as the control. After 3 days of treatment, cells were collected as previously reported [8]. In total, 50 µg/protein per sample were subjected to electrophoresis in 10% SDS-PAGE and transferred to nitrocellulose membranes. For immunodetection, the primary mouse monoclonal antibodies anti-aromatase (1:200), anti-ERα (1:200), and anti-AR (1:200; Santa Cruz Biotechnology, Santa Cruz, CA, USA), and the primary rabbit monoclonal antibodies anti-phospho-p42/44 (Thr202/Tyr204), anti-p42/44, anti-phospho-AKT (Ser463), and anti-AKT (Cell Signaling Technology Inc., Boston, MA, USA) were used. As secondary antibodies, the goat anti-mouse (1:2000) and the goat anti-rabbit (1:2000) antibodies (Thermo Fisher, Waltham, MA, USA) were used. A mouse monoclonal anti-β-actin antibody (1:500; Santa Cruz Biotechnology, Santa Cruz, CA, USA) was used to control loading variations. The membranes were further exposed to a chemiluminescent substrate WesternBright^TM^ ECL (Advansta Inc., Menlo Park, CA, USA) and the immunoreactive bands were visualized with a ChemiDoc™ Touch Imaging System (BioRad Laboratories, Melville, NY, USA). At least three independent experiments were performed for each protein. The protein expression obtained for treated cells was standardized in relation to protein expression of control.

### 2.5. RNA Extraction and qPCR Analysis

MCF-7aro cells were seeded in 6-well plates (7.5 × 10^5^ cell/mL) and treated with the AIs (10 µM), CBD (1 and 5 µM), and their combinations in the presence of 1 nM of T or E_2_ for 3 days. After treatment, cells were lysed and the RNA was collected as previously described [43]. Total RNA was quantified using the NanoDrop ND-1000 Spectrophotometer (NanoDrop Technologies, Inc., Wilmington, DE, USA). GRiSP Xpert cDNA Synthesis Mastermix (GRiSP Research Solutions, Porto, Portugal) was employed to obtain cDNA, which was further amplified using GRiSP Xpert Fast SYBR (GRiSP Research Solutions, Porto, Portugal), in the MiniOpticon Real-Time PCR Detection System (Bio-Rad Laboratories), as previously reported [12]. The sequences of the primers and the respective annealing temperatures are listed in Table 1. The housekeeping gene was β-Actin and the fold change in gene expression was calculated using the 2^−ΔΔCt^ method [44]. At least three independent experiments were performed for each gene. The mRNA transcript levels of treated cells were normalized in relation to the mRNA transcript levels of control.

### 2.6. siRNA Transfection

The siPORT NeoFX transfection agent (Gibco Invitrogen Co., Paisley, Scotland, UK) was used to perform the siRNA transfection, according to the manufacturer’s instructions. For each well, 5 μL of siPORT NeoFX transfection agent and 14 μL of siRNA negative control (scRNA; 10 μM; Santa Cruz Biotechnology, Santa Cruz, CA, USA) or of siRNA against ERα or AR (10 μM; Santa Cruz Biotechnology, Santa Cruz, CA, USA) were diluted in 100 μL of OPTI-MEM I medium (Gibco Invitrogen Co., Paisley, Scotland, UK). Both solutions were mixed and incubated at room temperature for 10 min. After trypsinization, MCF-7aro cells were resuspended in the mix of siRNA and transfection agent and then plated (1.5 × 10^5^ cells/mL) in 6-well plates. When adhered, the cells stimulated with T (1 nM) were treated with Exe (10 μM) plus CBD (5 μM) for 3 days. At least three independent experiments were performed.

### 2.7. ER and AR Transactivation Assays

The activity towards the human ER and AR was assessed as previously described [45], following the OECD Guidelines for the Testing of Chemicals, Tests No. 455 and 458, respectively. Both bioassays are based on stably transfected mammalian cell lines and are fully validated for a reliable detection of human ER and AR agonists and antagonists. Briefly, VM7Luc4E2 cells, expressing both α and β forms of the human ER, were kept in culture in DMEM without phenol red, supplemented with 4.5% CFBS, 1% penicillin/streptomycin, 2% L-glutamine, and 110 mg/mL sodium pyruvate for three days before the beginning of the experiments, and then plated (4 × 10^5^ cells/mL) in 96-well white plates. After adhesion, cells were exposed to Exe (1–10 µM), CBD (0.1–10 µM), or to their combination (5 µM CBD + 10 µM Exe) for 24 h, in the absence (ER agonism) or presence of 91.8 pM E_2_ or 1 nM T (ER antagonism). The activity was measured using the Steady-Glo^®^ Luciferase Assay System (Promega Corporation, Madison, WI, USA) in a multimode plate reader (EnSpire^®^, Perkin Elmer, Inc., Waltham, MA, USA). Viability was assessed using the CellTiter-Glo^®^ Luminescent Cell Viability Assay (Promega Corporation, Madison, WI, USA).

For assessment of AR activity, the AR-EcoScreen™ assay was performed, which uses Chinese hamster ovary (CHO-K1) cells expressing the human AR with a firefly luciferase reporter construct, and a renilla luciferase gene for viability estimation. Cells were seeded (9 × 10^4^ cells/mL) in DMEM/F12 without phenol red, containing 5% CFBS and 1% penicillin/streptomycin, in 96-well white plates, and exposed after cell adhesion to Exe (1–10 µM), CBD (0.1–10 µM), or to their combination (5 µM CBD + 10 µM Exe) for 24 h, in the absence or presence of 0.1 nM methyltrienolone (R1881; AbMole BioScience, Houston, TX, USA) for assessment of potential AR agonism and antagonism, respectively. AR activity and cell viability were assessed using the Dual-Glo^®^ Luciferase Assay System (Promega Corporation, Madison, WI, USA) in a multimode plate reader (EnSpire^®^, Perkin Elmer, Inc., USA). Data from four independent experiments were presented as fold change compared to control, which was set as 1. Data were normalized to control (cells not treated with Exe or CBD), which was set as 1.

T (781.2 pM–25.6 µM) and E_2_ (180 fM–367 nM) were tested as positive controls for ER agonism, while raloxifene (12.0 pM–24.5 nM; Biosynth Ltd., Berkshire, UK) was used as a positive control for ER antagonism. R1881 (7.8 pM–1 nM) and hydroxyflutamide (OHF; 4.1 nM–9 µM; Sigma-Aldrich Co., Saint Louis, MO, USA) were assessed as positive controls of AR agonism and antagonism, respectively. Stock solutions of T, E_2_, raloxifene, R1881, and OHF were prepared in 100% DMSO and stored at −20 °C. Dilutions were prepared freshly in medium before each experiment. The final concentration of DMSO in exposure medium was fixed at 0.06% for all conditions.

### 2.8. Statistical Analysis

Statistical analysis was performed with GraphPad Prism 8^®^ software (GraphPad Software, Inc., San Diego, CA, USA) and by the analysis of variance (ANOVA), followed by Bonferroni and Tukey post hoc tests for multiple comparisons (two-way ANOVA and one-way ANOVA, respectively). Values of *p* < 0.05 were considered statistically significant. All the data were expressed as the mean ± standard error of the mean (SEM). 

## 3. Results

### 3.1. Effects of CBD When Combined with AIs on Viability of Non-Tumorous Cells and Breast Cancer Cells

The effects of CBD (1 and 5 µM) when combined with AIs Ana, Let, or Exe (10 µM) on the viability of a sensitive ER^+^ breast cancer cell line, MCF-7aro cells, as well as on a non-tumor cell line, HFF-1 cells, were evaluated for 3 and 6 days. Of note, MCF-7aro cells were also stimulated with T (1 nM), which was used as proliferation inducing agent [8,10]. Additionally, the LDH assay for MCF-7aro cells was performed after 3 days of treatment.

**Figure 1:**
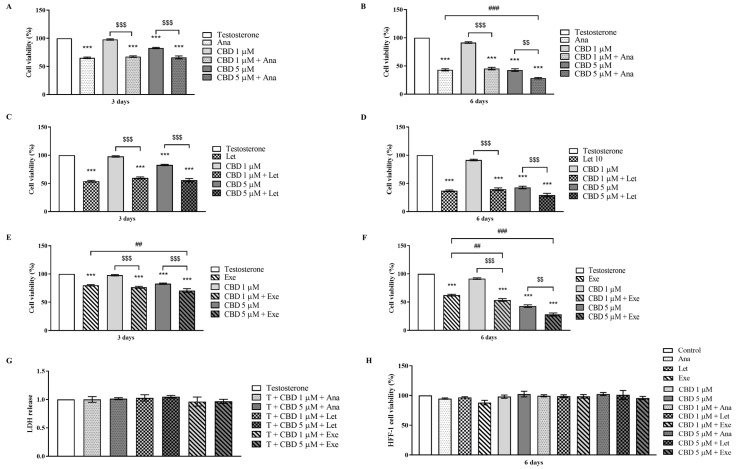
Effects of the AIs, Ana (**A**,**B**), Let (**C**,**D**), and Exe (**E**,**F**), CBD, and their combinations on MCF-7aro cell viability, as well as on the viability of HFF-1 cells (**H**). MCF-7aro cells were stimulated with T (1 nM) and treated with the AIs (10 µM), CBD (1 and 5 µM), or their combinations, over 3 or 6 days. Cells treated only with T were used as control, representing 100% of cell viability. After the 3 days of treatment, the LDH assay was also performed (**G**). The HFF-1 cells were subjected to the same concentrations of AIs and CBD for 6 days (**H**) and cells without any treatment were considered as control, representing 100% cell viability. The results are presented as mean ± SEM of at least three independent experiments performed in triplicate. Statistically significant differences between cells treated with compounds and T-treated cells (control) are expressed as *** (*p* < 0.001), while differences between the combinations and each AI alone are represented as ## (*p* < 0.01) and ### (*p* < 0.001), and the differences between the combinations and CBD as $$ (*p* < 0.01) and $$$ (*p* < 0.001).

The results presented in Figure 1A–F showed that all the combined treatments caused a significant (*p* < 0.001) reduction in MCF-7aro cell viability in comparison to the control (T-treated cells). In accordance with previous work [8,9,12,38], the AIs and CBD (5 μM) also affected cell viability. Furthermore, the effects induced by all the combinations were statistically significant (*p* < 0.01; *p* < 0.001) in relation to CBD alone. More importantly, for the combinations of CBD with Ana or Exe, but not with Let, a dose- and time-dependent reduction in MCF-7aro cell viability was observed when compared to the AIs. In fact, CBD only reduced significantly (*p* < 0.001) the cell viability of Ana-treated cells after 6 days of treatment, and at 5 µM CBD. In the case of Exe-treated cells, the significant (*p* < 0.01; *p* < 0.001) differences were noticed after 3 days for 5 µM CBD, and after 6 days for both CBD concentrations. In addition, as depicted in Figure 1G, none of the combinations induced LDH release, indicating that these treatments do not induce a loss of membrane integrity. Moreover, they also did not affect the viability of the non-tumor HFF-1 cells (Figure 1H).

### 3.2. Effects of CBD Plus AIs on Apoptotic Cell Death

**Figure 2:**
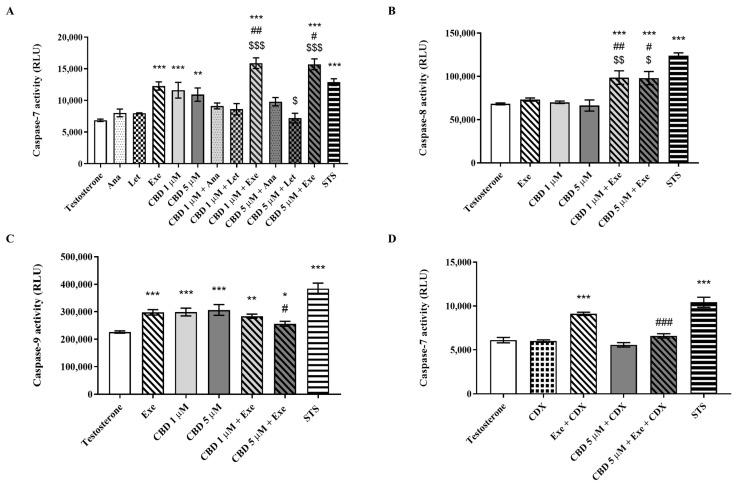
Analysis of MCF-7aro cell death. Effects of the AIs, Ana, Let, and Exe, as well as of CBD and their combinations on the activities of caspase-7 (**A**,**D**), caspase-8 (**B**), and caspase-9 (**C**). Cells were stimulated with T (1 nM) and treated with the AIs (10 µM), CBD (1 and 5 µM), or their combinations, for 2 days. Cells treated with Exe, CBD, and their combination were also treated with CDX (1 µM). Cells treated only with T were used as control, while STS-treated cells were considered as positive control. Statistically significant differences between treated cells and T (control) are expressed as * (*p* < 0.05), ** (*p* < 0.01) and *** (*p* < 0.001), while differences between the combinations and each AI alone are represented as # (*p* < 0.05), ## (*p* < 0.01) and ### (*p* < 0.001), and the differences between the combinations and CBD as $ (*p* < 0.05), $$ (*p* < 0.01) and $$$ (*p* < 0.001).

Considering the effects observed on cell viability, the possible involvement of apoptosis was further investigated. For that purpose, the activity of caspase-7, an effector caspase, as well as the activities of caspase-8 and -9 were evaluated in MCF-7aro cells treated for 2 days with CBD (1 and 5 µM) and/or AIs (10 µM) in the presence of T (1 nM). Here, it is important to note that caspase-3 was not evaluated because these cells do not express this caspase [46]. Contrary to the non-steroidal AIs Ana and Let, both Exe and CBD caused activation of caspase-7 (Figure 2A), which is in line with previous works [8,9,12,38]. More importantly, a significant (*p* < 0.01; *p* < 0.001) increase in caspase-7 activity was only observed for the combination of Exe plus CBD in relation to CBD and Exe (Figure 2A). For the combinations of CBD and Ana or Let, no effects on the activation of caspase-7 were detected (Figure 2A). Considering these results, the activities of the two initiator caspases, caspase-8 and -9, were only determined for the combinations of Exe plus CBD. A significant (*p* < 0.05; *p* < 0.01) increase was only observed for caspase-8 activity (Figure 2B,C) when compared with the isolated compounds, which caused no effect. In relation to caspase-9, although its activity was increased for the combination, no significant improvement in its activation was observed when compared to the isolated compounds.

### 3.3. Involvement of Aromatase in the Effects Induced by CBD Plus AIs

**Figure 3:**
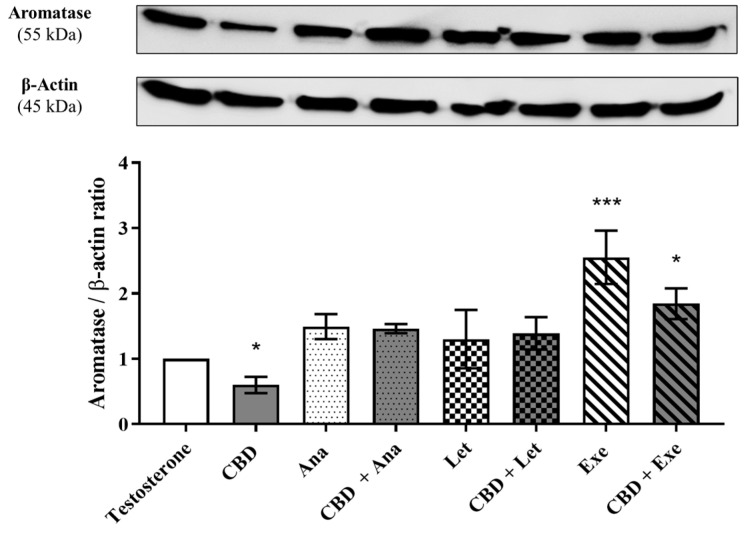
Effects of the AIs, Ana, Let, and Exe, as well as of CBD and their combinations on aromatase protein levels. MCF-7aro cells were stimulated with T (1 nM) and treated with the AIs (10 µM), CBD (5 µM), or their combinations, over 3 days. Cells treated only with T were used as control. A representative Western blot of aromatase and β-actin, as well as the densitometric analysis of aromatase expression levels after normalization with β-actin levels, used as loading control, are presented. Statistically significant differences between treated cells and T are expressed as * (*p* < 0.05) and *** (*p* < 0.001). The original Western blots are represented in Supplementary Figure S1A.

Considering that aromatase is one of the main therapeutic targets for the treatment of this type of tumor, and in order to understand the possible involvement of this enzyme on the effects induced by the combinations of CBD (5 μM) with AIs (10 μM) on MCF-7aro cells, their impacts on the protein levels of aromatase were investigated after 3 days of treatment. The results, as presented in Figure 3, demonstrated that CBD per se reduced the levels of aromatase (*p* < 0.05), while only Exe increased its levels (*p* < 0.001). None of the studied combinations affected the protein levels induced by the isolated AIs.

### 3.4. Involvement of ERα on the Effects Induced by CBD Plus AIs

**Figure 4:**
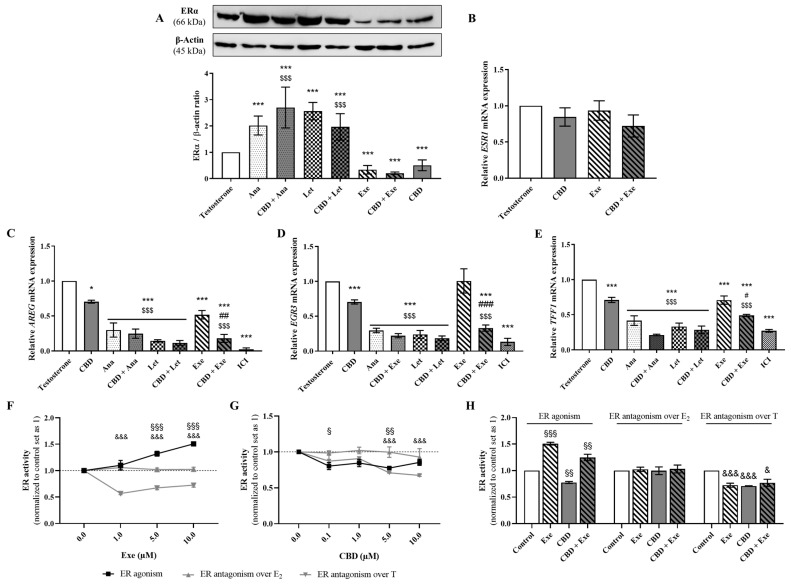
Involvement of ERα in the effects induced by the AIs, Ana, Let, and Exe, as well as of CBD and their combinations on breast cancer cells. MCF-7aro cells were stimulated with T (1 nM) and treated with the AIs (10 µM), CBD (5 µM) or their combinations for 3 days. Cells treated only with T were used as control. (**A**) A representative Western blot of ERα and β-actin, as well as the densitometric analysis of ERα expression levels after normalization with β-actin levels, used as loading control, are presented. (**B**–**E**) mRNA transcript levels for *ESR1* (**B**), *AREG* (**C**), *EGR3* (**D**), and *TFF1* (**E**) genes in relation to the housekeeping gene *β-actin*. (**F**–**H**) ER transactivation assay was performed in VM7Luc4E2 cells treated with Exe (0.1–10 µM; (**F**), CBD (0.1–10 µM; (**G**) and their combination (**H**) in the presence or absence of T (1 nM) or E_2_ (91.8 pM) over 24 h. Statistically significant differences between MCF-7aro cells treated with compounds and control (T) are expressed as * (*p* < 0.05) and *** (*p* < 0.001), while differences between the combinations and each AI alone are represented as # (*p* < 0.05), ## (*p* < 0.01) and ### (*p* < 0.001), and the differences between the combinations and CBD as $$$ (*p* < 0.001). For transactivation assays, the statistically significant differences between the control and cells treated with compounds but without T or E_2_ are expressed as § (*p* < 0.05), §§ (*p* < 0.01) and §§§ (*p* < 0.001), while differences between the control and cells treated with compounds in the presence of T are denoted by & (*p* < 0.05) and &&& (*p* < 0.001). The original Western blots are represented in Supplementary Figure S1B.

ERα is an important target for the development and survival of ER^+^ breast tumors [6]. Therefore, the effects of the combinations of CBD (5 µM) plus AIs (10 µM) on the protein levels of ERα, as well as on the transcription levels of *ESR1* and ERα-targeted genes, *AREG*, *EGR3*, and *TFF1,* were evaluated in MCF-7aro cells after 3 days of treatment. As expected, and previously reported, Ana and Let induced a significant (*p* < 0.001) increase in the protein levels of ERα [9], while Exe [9] and CBD [38] reduced (*p* < 0.001) its expression. Regarding the combinations, our results revealed that none induced alterations in ERα levels when compared to the isolated AIs (Figure 4A). As the combination of CBD plus Exe was the only one that maintained the reduction in ERα protein levels, the transcript levels of the *ESR1* gene were also studied. The results showed that neither CBD nor Exe nor their combination had a significant impact on the transcription of this gene (Figure 4B). On the contrary, in relation to ERα-targeted genes, and in accordance with our previous work [9], all the AIs significantly (*p* < 0.05; *p* < 0.001) reduced the transcription of *AREG* and *TFF1* genes (Figure 4C–E). However, and contrary to Ana and Let, which reduced the transcription of the *EGR3* gene, Exe did not affect its transcription when compared to the control (Figure 4D). Curiously, only when combined with Exe, CBD was able to significantly enhance the reduction in the transcription of the ERα-targeted genes AREG (*p* < 0.01), EGR3 (*p* < 0.001), and TFF1 (*p* < 0.05) when compared to Exe alone (Figure 4C–E). In fact, this combination significantly (*p* < 0.001) reduced the Exe-induced effect on the transcription of *EGR3* gene (Figure 4D).

Given that the combination of Exe plus CBD was the most promising, as it was the only one showing improved effects on all ERα-targeted genes, while also decreasing ERα protein levels, the ER activity was also evaluated. By using the VM7Luc4E2 cell line, the results revealed that Exe alone acted as an ER agonist (*p* < 0.001). In the presence of T, it displayed antagonistic properties (*p* < 0.001), while no effect was observed in the presence of E_2_ (Figure 4F). Like Exe, CBD in the presence of E_2_ had no effect on ER, whereas in the other studied conditions, it presented antagonistic activity (*p* < 0.05; *p* < 0.01; *p* < 0.001) (Figure 4G). When combined, Exe plus CBD exhibited agonistic effects on ER (*p* < 0.01; Figure 4H), but in the presence of T, this combination presented antagonistic properties (*p* < 0.05), while in the presence of E_2_, no effects were observed. Additionally, no significant effects on VM7Luc4E2 cell viability were observed, aside from 10 µM CBD (*p* < 0.05) (Supplementary Figure S2B).

### 3.5. Involvement of AR in the Effects Induced by CBD Plus AIs

**Figure 5:**
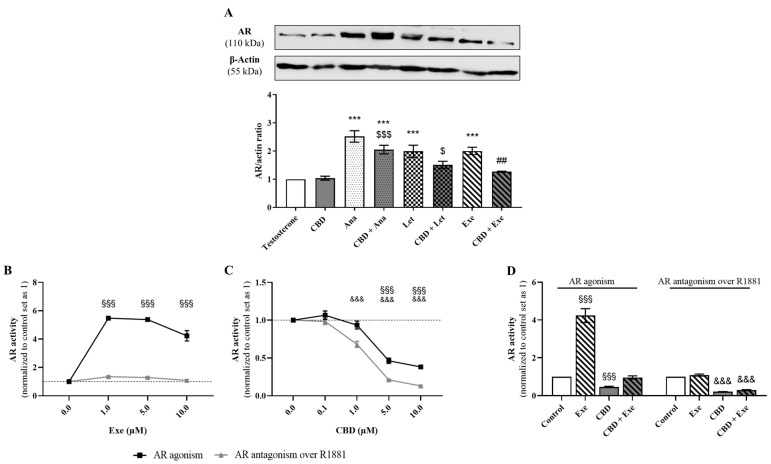
Involvement of AR in the effects of the AIs, Ana, Let, and Exe, as well as of CBD and their combinations on breast cancer cells. (**A**) AR protein expression levels were evaluated by Western blot in MCF-7aro cells stimulated with T (1 nM) and treated with the AIs (10 µM), CBD (5 µM) or their combinations for 3 days. Cells treated only with T were used as control. A representative Western blot of AR and β-actin, as well as the densitometric analysis of AR expression levels after normalization with β-actin levels, used as loading control, are presented. (**B**–**D**) AR transactivation assay was performed in the AR-EcoScreen™ cells treated with Exe (0.1–10 µM; B), CBD (0.1–10 µM; (**C**) and their combination (**D**) in the presence or absence of R1881 (0.1 nM) over 24 h. Statistically significant differences between MCF-7aro cells treated with compounds and control (T) are represented as *** (*p* < 0.001), while differences between the combinations and each AI alone are indicated as ## (*p* < 0.01) and the differences between the combinations and CBD as $ (*p* < 0.05) and $$$ (*p* < 0.001). For transactivation assays, the statistically significant differences between the control and cells treated with compounds but without R1881 are expressed as §§§ (*p* < 0.001), while differences between the control and cells treated with compounds in the presence of R1881 are denoted by &&& (*p* < 0.001). The original Western blots are represented in Supplementary Figure S1C.

Our group has previously demonstrated that AR plays an oncogenic and pro-survival role in Exe-treated MCF-7aro cells, increasing AR expression and activation [12], while in Ana- and Let-treated cells, this receptor has been associated with growth-inhibitory effects [9]. Taking this into account, in this study, the effects of the combination of CBD (5 µM) with AIs (10 µM) on AR expression levels were evaluated. The Western blot results, presented in Figure 5A, showed that in MCF-7aro cells, only the combination of Ana and CBD presented higher AR expression levels than the control, although to a lower extent than Ana alone. However, significant differences (*p* < 0.01) were detected between Exe-treated cells with or without CBD. Therefore, the activity towards AR was further assessed for this combination by performing the AR-EcoScreen™ assay. Our results showed that Exe per se has a significant (*p* < 0.001) agonistic effect on AR for all the concentrations studied (Figure 5B), while in the presence of R1881, a potent AR agonist, no significant effects were observed. On the contrary, 1 µM CBD exhibited significant (*p* < 0.001) antagonistic activity in the presence of R1881 (Figure 5C). Antagonism was also observed at higher concentrations (5 and 10 µM), though CBD decreased the cell viability of the AR-EcoScreen™ cell line in these conditions (Supplementary Figure S2E). Remarkably, the combination of Exe with CBD had a significant (*p* < 0.001) antagonistic effect on this receptor in the presence of 0.1 nM R1881 (Figure 5D). However, this effect may be masked by cytotoxicity, as cell viability was also significantly (*p* < 0.001) affected, though at a lower extent compared to antagonism (29.8% of AR activity, with 44.4% of cell viability) and should be thus regarded as a non-specific effect (Supplementary Figure S2F).

Considering that AR has an oncogenic role in Exe-treated MCF-7aro cells [12] and that Exe plus CBD causes a decrease in AR protein levels, the effects of this combination on caspase-7 activity in the presence of CDX (1 µM), an AR antagonist, were also explored in order to understand whether CBD has the ability to affect the pro-survival role of Exe. The results, presented in Figure 2D, show that when AR is blocked, Exe significantly increases (*p* < 0.001) caspase-7 activity, as previously reported [12], while when combined with CBD, this effect was completely reverted. In fact, a significant (*p* < 0.001) decrease in the activity of caspase-7 was detected between Exe and Exe plus CBD-treated cells when AR was blocked.

### 3.6. Crosstalk between ERα and AR

**Figure 6:**
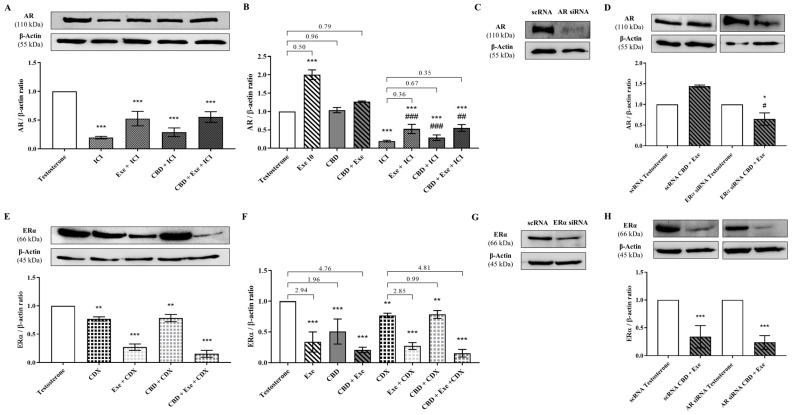
Crosstalk between ERα and AR for the combination of CBD with Exe in MCF-7aro cells. Effects of Exe (10 µM), CBD (5 µM), and their combination on AR protein levels, in the presence of ICI (100 nM; (**A**,**B**)) and after ERα knockdown (Figure (**C**,**D**)), and ERα protein levels, in the presence of CDX (1 µM; (**E**,**F**)) and after AR knockdown (Figure (**G**,**H**)). MCF-7aro cells were stimulated with T (1 nM) and treated with the Exe (10 µM), CBD (5 µM), or their combinations for 3 days. Cells treated only with T were used as control. For silencing, MCF-7aro cells were mixed with the siRNA desired and the transfection agent and further cultured in 6-well plates. In this case, cells treated with siRNA negative control (scRNA) were used as control, and (**C**,**G**), show that AR and ERα were silenced, respectively. A representative Western blot of AR, ERα, and β-actin, as well as the densitometric analysis of AR and ERα expression levels after normalization with β-actin levels, used as loading control, are presented. Statistically significant differences between treated cells and T are expressed as * (*p* < 0.05), ** (*p* < 0.01) and *** (*p* < 0.001), while differences between siRNA and scRNA, and ICI and ICI plus Exe, CBD or their combination are presented as # (*p* < 0.05), ## (*p* < 0.01) and ### (*p* < 0.001). The values presented in (**B**,**F**) are the ratio between the respective control and treatment. The original Western blots are represented in Supplementary Figure S1D–K.

Given the promising effects observed for the combination of Exe (10 μM) plus CBD (5 μM) on ERα and AR, and since we previously reported that Exe may exert its effects on breast cancer cells through a crosstalk between AR and ERα [9,12], this was further investigated for this combined treatment. For that purpose, the effects on the levels of AR in the presence of ICI (100 nM), an ERα down-regulator, and of ERα in the presence of CDX (1 µM), an AR antagonist, were evaluated. In addition, ERα and AR were silenced in Exe plus CBD-treated cells by performing siRNA for each receptor. The results presented in Figure 6A show that in the presence of ICI, all the treatments significantly (*p* < 0.001) decreased AR protein levels. More importantly, when comparing AR protein levels in the presence of ICI with those without ICI, significant (*p* < 0.01; *p* < 0.001) differences were observed (Figure 6B). Interestingly, it was possible to note a decrease in the ratios between control and treatments with and without ICI (Figure 6B). These results for Exe plus CBD-treated cells were corroborated with the knockdown of ERα (Figure 6C), where a statistically significant (*p* < 0.05) reduction in AR levels was detected between cells transfected with scRNA, and cells transfected with siRNA for ERα (Figure 6D). On the other hand, when AR is blocked by CDX, a significant (*p* < 0.01, *p* < 0.001) reduction in ERα protein levels was detected for all the treatments (Figure 6E). Moreover, the ratios of ERα protein levels when CDX is present were similar to those without CDX, except for CBD treatment which was lower in the presence of CDX (Figure 6F). A similar behavior was observed after the knockdown of AR (Figure 6G) in Exe plus CBD-treated cells, where no differences in ERα protein levels were detected between cells transfected with scRNA and cells transfected with siRNA for AR (Figure 6H).

### 3.7. Involvement of AKT and ERK1/2 Signaling Pathways in the Effects Observed for CBD Plus AIs

**Figure 7:**
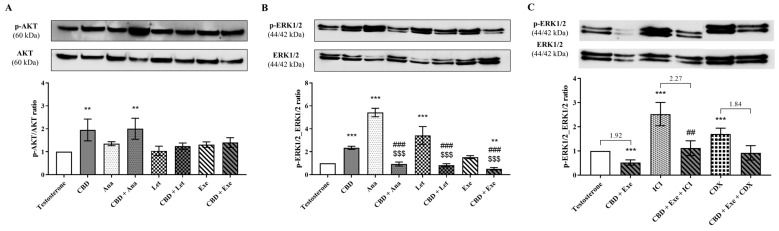
Effects of the AIs, Ana, Let, and Exe, as well as of CBD and their combinations on AKT (**A**) and ERK_1/2_ (**B**,**C**) signaling pathways. MCF-7aro cells were stimulated with T (1 nM) and treated with the AIs (10 µM), CBD (5 µM), or their combinations in the presence, or not, of ICI (100 nM) or CDX (1 µM) for 3 days. Cells treated only with T were used as control. A representative Western blot of p-p42/44 and p42/44, or of p-AKT and AKT, as well as densitometric analysis of p-p42/44 and p-AKT levels after normalization with p42/44 and AKT levels, respectively, is shown. Statistically significant differences between treated cells and T are expressed as ** (*p* < 0.01) and *** (*p* < 0.001), while differences between the combinations and each AI alone are represented as ## (*p* < 0.01) and ### (*p* < 0.001) and the differences between the combinations and CBD as $$$ (*p* < 0.001). The values presented in (**C**) are the ratio between the respective control and treatment. The original Western blots are represented in Supplementary Figure S1L–O.

To elucidate which signaling pathways may be involved in the anti-proliferative effects of AIs plus CBD combinations, MCF-7aro cells stimulated with T (1 nM) were treated with AIs (10 µM), with or without CBD (5 µM), for 3 days, and AKT and ERK_1/2_ activation was assessed. The results showed that CBD increased AKT phosphorylation, an effect abolished by its combination with Let and Exe, but not by Ana, which significantly (*p* < 0.01) increased AKT phosphorylation (Figure 7A). It can be hypothesized that the increase in AKT phosphorylation observed for the combination with Ana may be due to the effect induced by CBD per se, since no significant differences were found between CBD with or without Ana. On the other hand, the ERK_1/2_ signaling pathway was deeply affected by all the combinations (*p* < 0.001; Figure 7B) when compared to the individual compounds. In Ana- and Let-treated cells, an increase in ERK_1/2_ phosphorylation was observed, but this effect was totally reversed (*p* < 0.001) by their combination with CBD, with the levels of p-ERK_1/2_ being similar to the control. Contrary to the non-steroidal AIs, Exe per se did not affect this signaling pathway, though when combined with CBD, the levels of p-ERK_1/2_ were significantly (*p* < 0.01) lower than the control, and, more importantly, than Exe (*p* < 0.001).

Furthermore, taking into account the beneficial results observed for ERα and AR for the combination of Exe plus CBD, as well as the role of these receptors on the growth and survival of ER^+^ tumors, the involvement in ERK_1/2_ pathway activation was investigated in the presence of ICI (100 nM) or CDX (1 µM) for this combined treatment. Our results revealed that when ERα is downregulated, the combination of CBD with Exe causes a basal activation of ERK_1/2_ (Figure 7C), with the behavior being similar to Exe alone (Figure 7B). Therefore, ICI significantly (*p* < 0.01) reverted the decrease in ERK_1/2_ phosphorylation induced by the combination of Exe plus CBD (Figure 7C). On the other hand, when AR is blocked (CDX-treated cells), the activation of this signaling pathway was not significantly affected (Figure 7C). By comparing the ratios between controls and treatments, only an increase was observed for the combination in the presence of ICI, while for the combination in the presence of CDX, the ratio was similar to the cells only treated with Exe plus CBD.

### 3.8. Involvement of the Hormonal Environment on the Effects Induced by CBD Plus Exe

**Figure 8:**
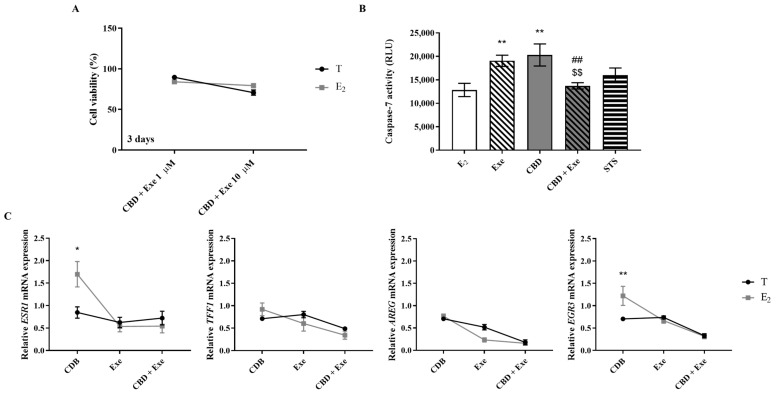
Effects of Exe or CBD and their combination on MCF-7aro cell viability (**A**), apoptosis (**B**), and mRNA transcript levels of *ESR1*, *TFF1*, *AREG*, and *EGR3* (**C**) in the presence of T (1 nM) or E_2_ (1 nM). Cells were stimulated with E_2_ or T and treated with the Exe (10 µM), CBD (1 and 5 µM), or their combinations for 2 or 3 days. Cells treated only with T or E_2_ were used as control, representing 100% of cell viability, and cells treated with STS were considered as positive control. mRNA transcript levels for *ESR1*, *AREG*, *EGR3*, and *TFF1* genes were analyzed in relation to the housekeeping gene *β-actin*. Statistically significant differences between treated cells and E_2_, or between E_2_- and T-treated cells are expressed as * (*p* < 0.05) and ** (*p* < 0.01), while differences between the combinations and Exe alone are represented as ## (*p* < 0.01), and the differences between the combinations and CBD as $$ (*p* < 0.01).

Considering that the most promising results regarding the combinations studied were those with Exe, we focused on its combination with CBD and investigated the involvement of the hormonal environment on those effects. For that, cells were treated with CBD (5 µM) and Exe (10 µM) in the presence of E_2_ (1 nM), for 2 or 3 days, and the effects on cell viability, caspase-7 activity, and ERα-targeted gene transcription were evaluated. As presented in Figure 8A, the effects on cell viability were similar to the ones observed in the presence of T. Curiously, unlike the isolated compounds, in the presence of E_2_, the combination did not induce an increase in the activity of caspase-7 (Figure 8B), a completely different behavior from that observed when cells are stimulated with T (Figure 2A). A significant (*p* < 0.01) reversion of the effects was observed between the combined and the isolated treatments.

Finally, when comparing the effects of the combination of CBD plus Exe on the transcription of ERα-targeted genes, as well as on *ESR1*, in the presence of T or E_2_, the only statistically significant differences observed were for CBD; it induced an increase in the transcription of *ESR1* (*p* < 0.05) and *EGR3* (*p* < 0.01) in the presence of E_2_ (Figure 8C).

## 4. Discussion

Despite the clinical success and effectiveness of the AIs applied in the clinic, nowadays, their use per se presents some limitations, which are mainly related to the development of endocrine resistance. In order to improve ER^+^ breast cancer treatment, a combination of endocrine therapies with other compounds, such as CDK4/6 inhibitors, mTOR inhibitors, PI3K inhibitors, and AR antagonists, are under investigation. In fact, the use of CDK4/6 inhibitors in combination with AIs or fulvestrant is now being suggested as the standard therapy for ER^+^ advanced breast cancer patients [4,13,14,15,16]. However, despite their success, they induce adverse side effects [7,16,17,18,19]. Considering this, in the current study, we investigated whether CBD, as an adjuvant therapy, could improve the effects of the AIs currently under clinical use. Previous work from our group showed that CBD, in MCF-7aro cells, induced cell cycle arrest at the G_0_/G_1_ phase, decreased aromatase expression, down-regulated ERα expression, and up-regulated ERβ expression, thus being able to modulate the three main targets responsible for ER^+^ breast cancer development and progression [38].

In this study, our results demonstrate that CBD combined with Ana or Exe was more effective than the isolated compounds in reducing the viability of breast cancer cells, with the combination of CBD plus Exe being the most promising one. In addition, it was demonstrated that this latter combination also induced a higher increase in the activity of the effector caspase-7 than Exe alone. Moreover, although this combination activated caspase-9 on the same proportion as Exe, contrary to this AI, it was able to activate caspase-8. This indicates that the results on cell viability observed for the combination of Exe plus CBD may occur by the promotion of apoptosis through the involvement of the mitochondrial pathway and caspase-8 activation, which is in line with our previous results for Exe [8] and CBD [38]. Moreover, a cross-talk between this pathway and caspase-8 was also observed for Exe in sensitive and resistant breast cancer cells when autophagy [8,42], PI3K [42], and AR [12] were targeted. The combination of the non-steroidal AIs with CBD did not potentiate cell death by apoptosis, which may explain the lack of significant effects observed in the cell viability studies.

Considering that the main targets for this subtype of cancer are aromatase, ER, and AR, the effects of CBD plus AIs were investigated in these targets. In relation to aromatase, it should be firstly noted that in MCF-7aro cells, both CBD [38] and AIs [9,47] inhibit aromatase, and that after 8h of incubation, CBD impairs the synthesis of aromatase [38], while Exe is the only AI able to induce aromatase degradation [9,48,49,50]. Our results showed that all combinations increased the protein levels of this enzyme. The results obtained with Exe, after 3 days of treatment, are in accordance with those previously described by Wang X. et al. [48]. However, the effects of Ana, Let, and CBD on aromatase expression were reported here for the first time, for this time of exposure. On the other hand, although CBD per se decreased the expression of aromatase, its combination with AIs did not affect the effects induced by AIs. This may be explained by the greater affinity of the AIs for the aromatase binding site than CBD.

In relation to ER, previous studies from our group revealed that Ana and Let induce a significant increase in the protein levels of ERα [9], while Exe [9] and CBD [38] significantly reduce its levels in breast cancer cells. Although our results corroborated these observations, they also showed that the combinations did not affect ERα expression. Nevertheless, it should be noted that no alterations in *ESR1* gene transcription were observed for the combination of Exe plus CBD, with this behavior being similar to that of the isolated compounds. Despite that, all the combinations decreased the transcription of the ERα-targeted genes, *AREG*, *EGR3*, and *TFF1*, in a similar way to the AIs. However, the reduction observed for Exe plus CBD was higher than with Exe, which highlights the beneficial effect of this combination. In fact, it should be pointed out that, as previously described [9], and despite inducing a reduction in ERα expression, Exe does not decrease the transcription of the *EGR3* gene that is considered the bone fide target of ERα [51]. CBD may circumvent the weak estrogen-like effect of Exe [9,52] by preventing the transcription of the *EGR3* gene. Moreover, Exe displayed a clear agonistic activity over ER, while CBD acted as an antagonist of this receptor. The behavior verified for Exe reinforces previous studies from our group where, in the absence of hormonal influence, Exe caused a strong activation of ERα denoted by an increase in the transcription of ERα-target genes [9], confirming the weak estrogen-like effects of Exe. However, in the presence of T, the agonistic activity of Exe was reverted and this AI acted as an ER antagonist, demonstrating that the anti-cancer properties are dependent on the hormonal environment [9,52]. On the contrary, CBD preserved its antagonistic behavior. These data corroborate our previous work with CBD [38], where a mechanism of action similar to the selective ER down-regulator (SERD) ICI was suggested [53,54,55]. From a clinical point of view, this is very important, since the development of novel drugs targeting ERα and cannabinoid receptors may be relevant for future personalized cancer therapies [36,56,57,58]. Our data also demonstrated that the combination exerts agonistic effects on ER under hormone-depleted conditions, suggesting that, in this case, the effects of Exe on this receptor override those of CBD. In the presence of T, the combination displayed an antagonistic behavior, being similar to the isolated compounds. These data reinforce the therapeutic importance of this combination and the ability of CBD to modulate the function of ERα, acting as a SERD, which overcomes the weak estrogen-like effect pointed to Exe.

Regarding AR, the combination of CBD plus Exe triggered interesting effects. As previously referred to, the three AIs increased AR protein levels, with Ana and Let displaying growth-inhibitory properties [9] and Exe displaying pro-tumorigenic properties [12]. On the other hand, CBD had no effects on AR protein levels in breast cancer cells [38]. However, in this study, contrary to the combinations with Ana and Let, the combination of CBD plus Exe caused a significant decrease in AR expression levels when compared to Exe alone. This result is especially important since it may reduce the pro-oncogenic potential of Exe in breast cancer cells. On the contrary, as AR for the non-steroidal AIs presents a pro-death role [9], the reduction in its expression induced by CBD may not be beneficial, which may explain why their combination with CBD did not improve the anti-proliferative effects of these AIs on breast cancer cells. Interestingly, when evaluating the activity of Exe, CBD, and their combination towards AR, our results confirmed the agonistic effect of Exe, and showed that CBD and the combination act as AR antagonists. Thus, and contrary to what was verified for ER, CBD is the main driver of the effects of the combination on AR. In addition, the switch in the AR function, when CBD is added to Exe-treated cells, was confirmed by the evaluation of the caspase-7 activity in the presence of the AR antagonist CDX. In these conditions, no increment was observed, and even a reduction in caspase-7 activity was detected in relation to Exe, meaning that the increase in caspase-7 activity after CBD treatment is dependent on AR. Therefore, it is possible to conclude that the action of CBD on AR overrides that of Exe, with CBD having a pro-death effect through AR, thus reverting the pro-survival role of AR in Exe-treated cells [12].

In addition, the results point to a regulation/modulation of AR by ERα, as in the presence of the ERα down-regulator ICI, or after the knockdown of ERα, a more pronounced reduction in AR levels was observed, even lower than that of the control. Curiously, the same reduction was also detected for Exe or CBD after treatment with ICI. This demonstrates that for Exe and CBD, the AR overexpression is also modulated by Erα, but, in the case of CBD, this AR regulation guarantees the pro-death role of this receptor. On the contrary, in the presence of the AR antagonist CDX or after the knockdown of AR, no differences in the ERα expression levels were observed. This crosstalk is corroborated by the analysis of the survival signaling pathways, AKT and ERK_1/2_. The results showed that the effects induced by all the AIs, CBD, or their combinations were not mediated through AKT signaling. In the case of the ERK_1/2_ pathway, Exe plus CBD led to a reduction in the activation of this pathway that was even lower than the control. This effect was influenced by ERα and not by AR. Thus, the inhibition of the activation of the ERK_1/2_ pathway may be mediated by the ERα effects induced by the combination of Exe plus CBD, which is responsible for its anti-proliferative effects.

**Figure 9:**
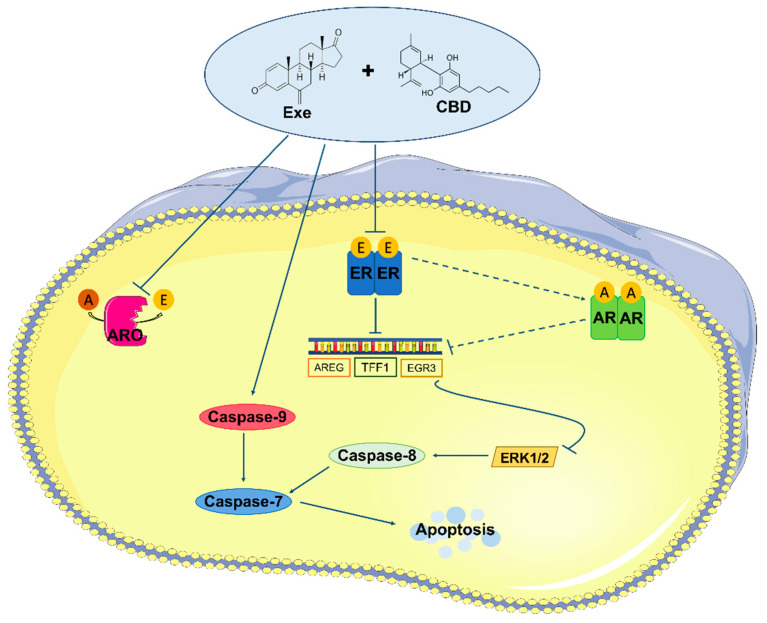
Schematic representation of the effects induced by Exe plus CBD in MCF-7aro cells. This combination compromises the binding of estrogens, produced by aromatase, to ERα, decreasing ERα protein levels and the transcription of some ERα-targeted genes, including *EGR3*. The reduction in *EGR3* levels inhibits the activation of ERK_1/2_ signaling pathway, which prevents the inhibition of pro-caspase-8, that is converted to caspase-8, and, together with caspase-9, activates caspase-7, promoting apoptosis. Moreover, the effects of the combination on ERα modulate AR through an unknown crosstalk, contributing to the decreased *EGR3* levels, avoiding the estrogen-like effect. Exe: Exemestane; CBD: cannabidiol; A: androstenedione; E: estrogens; ARO: aromatase; ER: estrogen receptor; AR: androgen receptor.

Still regarding ERK_1/2_ signaling, Mandal et al. demonstrated that the phosphorylation of pro-caspase-8 by ERK_1/2_ prevented its cleavage into caspase-8 and, therefore, the apoptotic cell death mediated by cell death receptors [59]. This is in accordance with our results, as a decrease in ERK_1/2_ activation in cells treated with Exe plus CBD was observed and, at the same time, an activation of caspase-8. Therefore, it can be hypothesized that by reducing ERα signaling, Exe plus CBD inhibits the activation of the ERK_1/2_ pathway. This does not prevent the conversion of the inactive pro-caspase-8 into active caspase-8, which, consequently, together with caspase-9, activates caspase-7, thus enhancing apoptosis (Figure 9). Moreover, it is important to remember the constant crosstalk between ERα and AR in ER^+^ breast cancer, which may explain the dependence of caspase-7 activity on AR levels (Figure 9). On the other hand, Exe decreases the expression of ERα, as well as the transcription of its targeted genes, except for *EGR3* levels which remain stable. This effect was already attributed to AR [9], whose levels are overexpressed [12]. Thus, the increase in AR levels is enough to maintain the levels of *EGR3* transcripts, the most important gene related to estrogenic effects. As previously discussed, CBD per se does not affect AR protein levels [38], but, when combined with Exe, its effects overlap with Exe’s effects, reducing AR levels, as well as the transcript levels of *EGR3*. With this, the estrogenic effect is fully compromised and the activation of ERK_1/2_ signaling is reduced, favoring the promotion of apoptosis. Therefore, the estrogenic effect is suggested to be the main target affected by the action of Exe plus CBD combination therapy, which is supposed to, ultimately, drive the anti-proliferative effects.

An important aspect that may influence potential tumor therapies is the hormonal microenvironment. In relation to CBD, there was an increase in the transcription of *ESR1* and *EGR3* genes in the presence of E_2_ compared to T. These results together with those from the ER transactivation assay suggest that CBD may bind to the active site of ERα, competing with E_2_ for the binding to the receptor, since in the presence of E_2_ the CBD antagonism and the inhibition of the transcription of ERα-target genes is lost. Additionally, in relation to the combination of Exe plus CBD, the promotion of apoptosis was compromised in an E_2_-enriched environment, as no caspase-7 activation was detected. Moreover, no effects on cell viability and on transcript levels of ERα and its target genes were noted between T- and E_2_-treated cells. It is also important to note that in the presence of E_2_, the antagonistic activity of the combination is lost. Therefore, altogether, these results suggest that the apoptotic actions of Exe plus CBD, as well as the antagonistic effect on ER, are lost in an E_2_-enriched environment, compromising the beneficial effects of this possible therapeutic approach. This indicates that, in contrast to what was suggested for Exe per se [9], Exe plus CBD should not be applied in the early stages of ER^+^ breast tumors where the levels of E_2_ are still high.

## 5. Conclusions

This study reinforces the potential of cannabinoids, particularly of CBD, to exert anti-cancer actions in ER^+^ breast cancer. This non-psychoactive phytocannabinoid has not only shown promising effects, but it is also able to potentiate the pro-cell death effects of the steroidal AI used in clinic, Exe, improving its efficacy. Indeed, this combination may potentially be more attractive than current approaches that combine endocrine therapy with other agents, as these have several side effects and limited efficacy. In contrast, it should be pointed out that in breast cancer patients treated with the non-steroidal AIs, Ana and Let, their combination with CBD does not appear to have beneficial effects from a therapeutic point of view. Thus, this study opens up a new potential and promising line of research for the improvement of ER^+^ breast cancer therapy, mainly for patients undergoing Exe treatment. Therefore, a new and appealing therapeutic approach with CBD as an adjuvant therapy was highlighted.

## Figures and Tables

**Table 1:** Primer sequences and annealing temperatures for housekeeping and target genes.

Symbol	Primers	Annealing Temperature
*AREG*	Forward: 5′-TGTCGCTCTTGATACTCGGC -3′ Reverse: 5′-ATGGTTCACGCTTCCCAGAG -3′	56 °C
*EGR3*	Forward: 5′-GACTCCCCTTCCAACTGGTG-3′ Reverse: 5′- GGATACATGGCCTCCACGTC-3′	56 °C
*TFF1*	Forward: 5′-GTGGTTTTCCTGGTGTCACG-3′ Reverse: 5′-AGGATAGAAGCACCAGGGGA-3′	55 °C
*β-Actin*	Forward: 5′-TACAGCTTCACCACCACAGC-3′ Reverse: 5′- AAGGAAGGCTGGAAGAGAGC-3′	55 °C

## Data Availability

The data can be shared up on request.

## Supplementary Materials

The following supporting information can be downloaded at https://www.mdpi.com/article/10.3390/cancers15092517/s1. Figure S1: The original Western Blot images for the Western Blots presented in the manuscript can be found. Figure S2: The effects of Exe or CBD and their combination on the viability of VM7Luc4E2 cells and AR-EcoScreen™ cells are presented.
