# Supplementary material for: Cannabidiol as a Promising Adjuvant Therapy for Estrogen Receptor-Positive Breast Tumors: Unveiling Its Benefits with Aromatase Inhibitors
Source: Cancers (Basel). 2023 Apr 27;15(9):2517. doi: 10.3390/cancers15092517 (PMC10177097; doi:10.3390/cancers15092517)
Supplement: Supplementary file 1 [file cancers-15-02517-s001.zip › Supplementary figures legends.pdf]

Supplementary Figure S1: Original Western Blot images of the Western-Blots presented in the manuscript. All the experiments were performed using β-actin or the full form of the protein under study as a loading control. **(A)** Western Blot of aromatase shown on figure 3. The order of the samples in the gel was T, CDB (5 μM), Ana, CDB (5 μM) + Ana, Let, CDB (5 μM) + Let, Exe, CDB (5 μM) + Exe. **(B)** Western Blot of ERα shown on figure 4A. The order of the samples in the gel was T, Ana, CDB (5 μM) + Ana, Let, CDB (5 μM) + Let, Exe, CDB (5 μM) + Exe, CDB (5 μM). **(C)** Western Blot of AR shown on figure 5A. The order of the samples in the gel was T, CDB (5 μM), Ana, CDB (5 μM) + Ana, Let, CDB (5 μM) + Let, Exe, CDB (5 μM) + Exe. **(D)** Western Blot of AR shown on figure 6A. The order of the samples in the gel was T, ICI, Exe + ICI, CBD + ICI, CBD + Exe +ICI. **(E)** Western Blot of AR shown on figure 6C. The order of the samples in the gel was X, X, X, X, scRNA, siRNA, AR. **(F)** Western Blot of AR shown on figure 6D. The order of the samples in the gel was X, X, scRNA AR T, scRNA CBD + Exe. **(G)** Western Blot of AR shown on figure 6D. The order of the samples in the gel was X, X siRNA AR T, siRNA ERα CBD + Exe, X, X. **(H)** Western Blot of ERα shown on figure 6E. The order of the samples in the figure was T, CDX, Exe + CDX, CBD + CDX, CBD + Exe + CDX. (I) Western-Blot of ERα shown on figure 6G. The order of the samples in the figure was X, X, X, X, scRNA, siRNA ERα. **(J)** Western Blot of ERα shown on figure 6H. The order of the samples in the figure was X, X, scRNA T, scRNA CBD + Exe. **(K)** Western Blot of ERα shown on figure 6H. The order of the samples in the figure was X, X, siRNA AR T, siRNA AR CBD + Exe, X, X. **(L)** Western Blot of p-AKT shown on figure 7A. The order of the samples in the gel was T, CDB (5 μM), Ana, CDB (5 μM) + Ana, Let, CDB (5 μM) + Let, Exe, CDB (5 μM) + Exe. **(M)** Western Blot of p-ERK$_{1/2}$ shown on figure 7B. The order of the samples in the gel was T, CDB (5 μM), Ana, CDB (5 μM) + Ana, Let, CDB (5 μM) + Let, Exe, CDB (5 μM) + Exe. **(N)** Western Blot of p-ERK$_{1/2}$ shown on figure 7C. The order of the samples in the gel was T, CDB + Exe, ICI, CBD + Exe + ICI, X, X, X, X. **(O)** Western Blot of p-ERK$_{1/2}$ shown on figure 7C. The

order of the samples in the gel was X, X, X, X, X, X, CDX, CBD + Exe + CDX. X represents samples of unrelated experiments.

Supplementary Figure S2: Effects of Exe or CBD and their combination on the viability of VM7Luc4E2 cells (**A-C**) and AR-EcoScreen™ cells (**D-F**). Cells were treated with Exe (0.1 – 10 μM; **A and D**), CBD (0.1 – 10 μM; **B and E**) and their combination (**C and F**) in the presence or absence of T (1 nM), E_2 (91.8 pM) and R1881 (0.1 nM), after 24h of incubation. Data were normalized to control (cells not treated with Exe or CBD), which was set as 1. The results are presented as mean ± SEM of four independent experiments performed in triplicate. Statistically significant differences between cells treated without T, E_2 or R1881 and the control are expressed as * ($p < 0.05$) and *** ($p < 0.001$), while the differences between treated cells in the presence of R1881 and the control are indicated by &&& ($p < 0.001$).
