# Supplementary figures and images for: Cannabidiol as a Promising Adjuvant Therapy for Estrogen Receptor-Positive Breast Tumors: Unveiling Its Benefits with Aromatase Inhibitors
Source: Cancers (Basel). 2023 Apr 27;15(9):2517. doi: 10.3390/cancers15092517 (PMC10177097; doi:10.3390/cancers15092517)

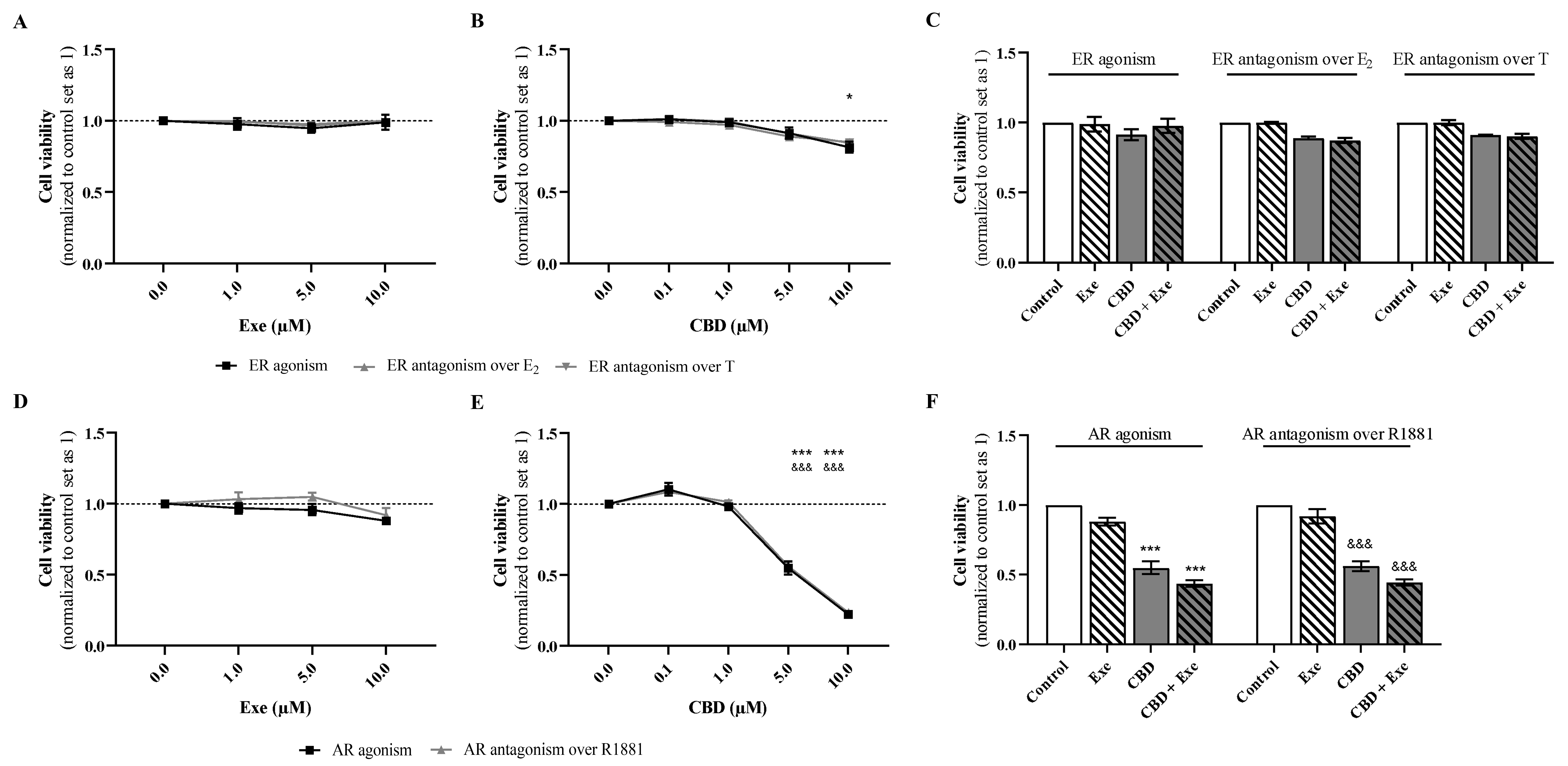

Supplement: Supplementary file 1 [file cancers-15-02517-s001.zip › Suplementary Figure S2.jpg]

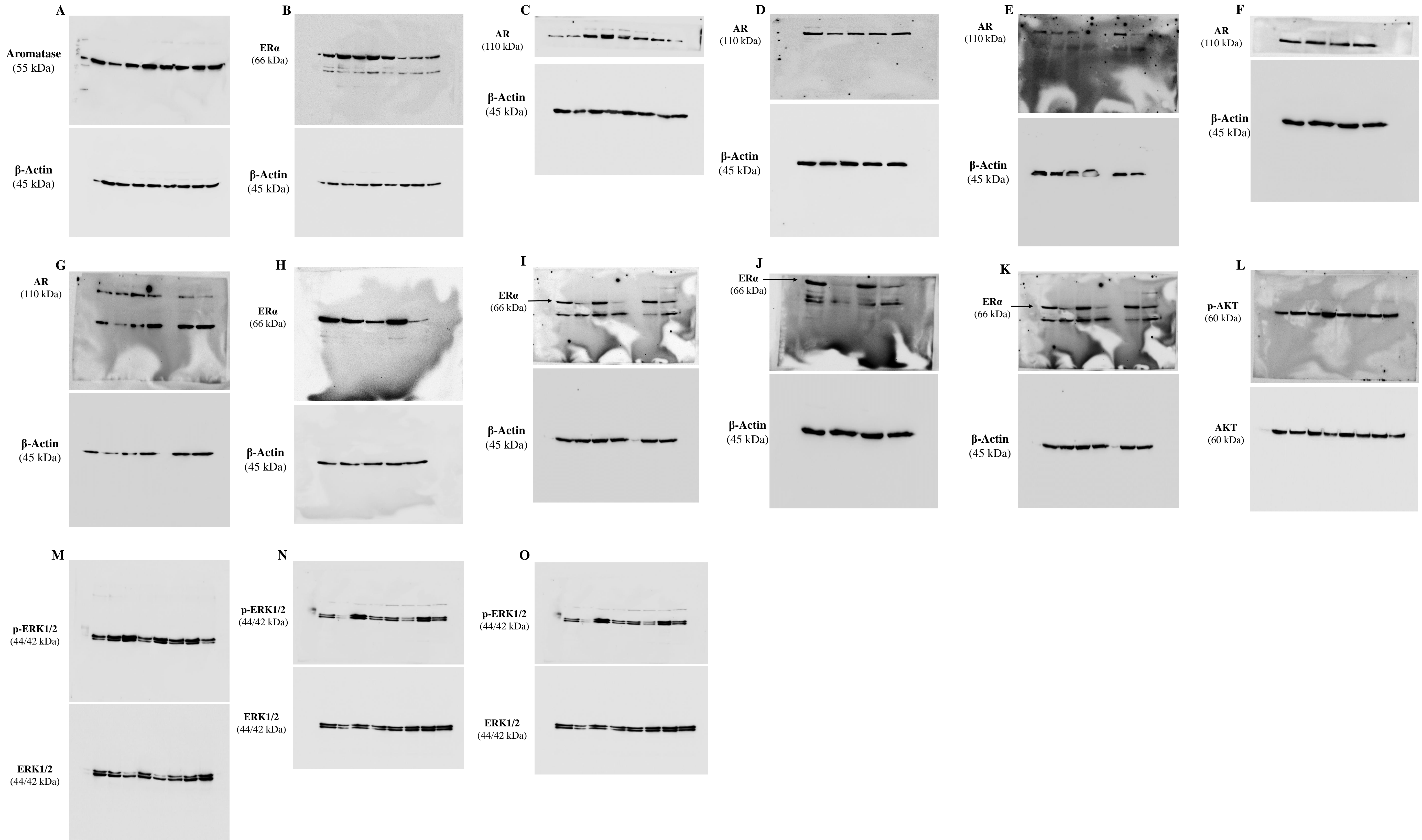

Supplement: Supplementary file 1 [file cancers-15-02517-s001.zip › Supplementary Figure S1.pdf]
